# Pigment Epithelium-Derived Factor Plays a Role in Alzheimer’s Disease by Negatively Regulating Aβ42

**DOI:** 10.1007/s13311-018-0628-1

**Published:** 2018-05-07

**Authors:** Mao Huang, Weiwei Qi, Shuhuan Fang, Ping Jiang, Cong Yang, Yousheng Mo, Chang Dong, Yan Li, Jun Zhong, Weibin Cai, Zhonghan Yang, Ti Zhou, Qi Wang, Xia Yang, Guoquan Gao

**Affiliations:** 1 Program of Molecular Medicine, Affiliated Guangzhou Women and Children’s Hospital, Zhongshan School of Medicine, Sun Yat-sen University, Guangzhou, China; 2 Department of Biochemistry, Zhongshan School of Medicine, Sun Yat-sen University, 74 Zhongshan 2nd Road, Guangzhou, 510080 China; 3 Institute of Clinical Pharmacology, Guangzhou University of Chinese Medicine, Guangzhou, China; 4 Guangdong Province Key Laboratory of Brain Function and Disease, Zhongshan School of Medicine, Sun Yat-sen University, Guangzhou, China; 5 China Key Laboratory of Tropical Disease Control (Sun Yat-sen University), Ministry of Education, Guangzhou, China; 6 Guangdong Engineering & Technology Research Center for Gene Manipulation and Biomacromolecular Products, Sun Yat-sen University, Guangzhou, China

**Keywords:** Alzheimer’s disease, Pigment epithelium-derived factor, Aβ42, Presenilin-1

## Abstract

Alzheimer’s disease (AD) is the most common cause of dementia. Pigment epithelium-derived factor (PEDF), a unique neurotrophic protein, decreases with aging. Previous reports have conflicted regarding whether the PEDF concentration is altered in AD patients. In addition, the effect of PEDF on AD has not been documented. Here, we tested serum samples of 31 AD patients and 271 normal controls. We found that compared to PEDF levels in young and middle-aged control subjects, PEDF levels were reduced in old-aged controls and even more so in AD patients. Furthermore, we verified that PEDF expression was much lower and amyloid β-protein (Aβ)42 expression was much higher in senescence-accelerated mouse prone 8 (SAMP8) strain mice than in senescence-accelerated mouse resistant 1 (SAMR1) control strain mice. Accordingly, high levels of Aβ42 were also observed in PEDF knockout (KO) mice. PEDF notably reduced cognitive impairment in the Morris water maze (MWM) and significantly downregulated Aβ42 in SAMP8 mice. Mechanistically, PEDF downregulated presenilin-1 (PS1) expression by inhibiting the c-Jun N-terminal kinase (JNK) pathway. Taken together, our findings demonstrate for the first time that PEDF negatively regulates Aβ42 and that PEDF deficiency with aging might play a crucial role in the development of AD.

**Electronic supplementary material:**

The online version of this article (10.1007/s13311-018-0628-1) contains supplementary material, which is available to authorized users.

## Introduction

Alzheimer’s disease (AD) is the most common cause of dementia among the elderly, affecting up to 35.6 million people worldwide [1]. Unfortunately, a cure for AD has not been identified yet. Senile plaques (SPs) composed of ~ 4-kDa amyloid β-protein (Aβ) fibrils are generally considered as the upstream causative factor as well as a major therapeutic target of AD [2, 3]. Although Aβ42 comprises less than 10% of secreted Aβ species, Aβ42 is highly prone to aggregation and is the major form of Aβ deposited in SPs [4]. Furthermore, besides extracellular Aβ42, intracellular Aβ42 aggregation is an early event that may precede extracellular SP formation and contribute to neuronal damage [5–7]. Nevertheless, the reason for Aβ42 aggregation in AD patients is not well understood.

Physiologically, amyloid precursor protein (APP) is cleaved first by α-secretase and subsequently by γ-secretase to release the p3 peptide, which is considered nonamyloidogenic [2]. Pathologically, APP undergoes site-specific proteolysis first by β-secretase 1 (BACE1) and is subsequently cleaved by γ-secretase at multiple sites, releasing the small peptides Aβ40 and Aβ42 [8]. Increased expression of presenilin-1 (PS1), an essential component of γ-secretase [9, 10], contributes to Aβ42 generation [9, 11] and ultimately leads to AD [12, 13]. However, the regulation of PS1 has not been fully described.

Pigment epithelium-derived factor (PEDF), a 50-kDa unique neurotrophic and neuroprotective protein, is widely expressed in the nervous system. PEDF protects neurons from insults such as quinolinic acid excitotoxicity, glutamate excitotoxicity, and oxidative damage [14, 15]. Furthermore, PEDF can protect retinal neurons from damage caused by transient ischemic reperfusion [16], and ischemic brain damage can be attenuated by the overexpression of PEDF [17]. According to previous studies, changes in PEDF concentrations observed in AD patients are controversial [18–21]. Moreover, the effect of PEDF on AD has not been verified.

Our previous studies focused on the effect of PEDF in diabetes and cancer. Interestingly, both diabetes and cancer are age-related diseases [22–25]. PEDF levels are decreased in the retina of diabetic patients and in multiple cancers according to our previous reports [26–28]. It is well known that age is a major risk factor for AD, with a doubling of risk every 5 years in individuals over 65 years of age [29, 30]. Further studies have correspondingly observed intraneuronal Aβ42 deposits in aged monkeys and canines [31, 32], suggesting that intraneuronal Aβ42 accumulation may be a fundamental pathology in the aged brain. Likewise, it has been shown that there is a decrease in PEDF expression with aging [33].

These findings have collectively led to the hypothesis that PEDF may be associated with AD. Therefore, changes in PEDF expression and the role and underlying mechanism of PEDF in AD patients and an animal model are systematically explored in the present study.

## Material and Methods

### Human Subjects

A total of 271 normal human samples were collected from the Guangzhou First People’s Hospital (*N* = 23), the Third Affiliated Hospital of Guangzhou Medical University (*N* = 65), Guangzhou Women and Children’s Medical Center (*N* = 24), the Third People’s Hospital of Zhongshan (*N* = 14), and the Guangzhou Blood Center (*N* = 145). The AD patient samples were all collected from the Third People’s Hospital of Zhongshan (*N* = 31). The collection was in strict agreement with the institutionally approved guidelines, and each participant gave written informed consent. The AD patients were clinically diagnosed according to *Diagnostic and Statistical Manual of Mental Disorders Fourth Edition Text Revision* (*DSM-IV-TR*) and the National Institute of Neurological and Communicative Disorders and Stroke and the Alzheimer’s Disease and Related Disorders Association (NINCDS-ADRDA) criteria. The clinical characteristics of the subjects are presented in Supplementary Table 1.

### Cell Culture

APP-PS1(M146L) CHO cells were purchased from Bailey Biological Technology Company, Shanghai. The antibiotic G418 (0.5 μg/ml, Sigma, Santa Clara, CA) was used for the generation of stable cell lines. The bEnd.3 cell line was purchased from Cellcook Company, Guangzhou. A well-differentiated PC12 cell line was obtained from the Cell Bank of the Chinese Academy of Sciences (Shanghai, China) with completed mycoplasma detection. All cells were maintained in DMEM supplemented with 10% fetal bovine serum (FBS). Penicillin and streptomycin were added to all cultures.

### Experimental Animals and Protocols

The research was carried out in strict accordance with the recommendations in the Guide for the Care and Use of Laboratory Animals of the National Institutes of Health. All animals were treated with regard to alleviation of suffering. The SAMP8 mice (7 months old) and the age-matched SAMR1 mice were purchased from Tianjin University of Traditional Chinese Medicine (Tianjin, China). The PEDF knockout (KO) mice were provided as a gift by Dr. S. J. Wiegand (Regeneron Pharmaceuticals, Inc., Tarrytown, NY) [34]. The SAMP8 mice were randomly divided into 2 groups with 12 animals per group. Recombinant glutathione S-transferase (GST)-PEDF (PEDF) was obtained as previously described [35]. One of the groups of mice was intraperitoneally injected with recombinant PEDF (4 mg/kg) daily for a month, and the other groups of SAMP8 and SAMR1 mice were simultaneously injected with the same volume of PBS as a control. The Morris water maze (MWM) was performed as follows: a behavioral test, latency experiment for 5 days, and the probe test on the 6th day.

### Morris Water Maze

The MWM setup included a circular, stainless steel tank (120 cm in diameter) filled with water (25 ± 1 °C) to a depth of 30 cm. The maze was divided into 4 virtual quadrants, and a platform painted white was placed in the center of the northeast quadrant, ~ 2 cm underwater. The platform remained in the same position throughout the experiment and was then removed from the pool during the probe test on the last day of the trials. Several distal extra-maze spatial cues were placed around the pool, which remained in the same position throughout the training and testing periods. The training trials were preceded by testing trials, in which the mice were allowed to explore the maze for 90 s from 4 different directions (east, south, west, and north). The mice were placed into the maze facing the edge in every trial. A trial was considered completed if the mouse had found the platform, climbed on to it and stayed there for 20 s. The mouse would also be placed on the platform for 20 s if it had not found the platform within 90 s. To examine the spatial reference memory of the animals, a probe test was carried out at 24 h after the last testing trial. During the probe test, each mouse was allowed to swim freely for 90 s without the platform. The tracking data from all trials were analyzed for a number of behavioral parameters using SMART software.

### Cellular Uptake of Recombinant Rluc-PEDF

Recombinant His-Renilla luciferase-PEDF (86 kDa) was expressed in *Escherichia coli* and purified with a His-Bind affinity column (17-5318-02, Novagen, Kenilworth, NJ) according to the manufacturer’s recommendations. The recombinant constructed plasmid map that we made is shown in Supplementary Fig. 1A. Recombinant protein expression was confirmed by Western blotting analysis using a rabbit antihuman PEDF polyclonal antibody that we made previously (data not shown). The bEnd.3 cells were seeded into 24-well plates at a concentration of 6 × 10^4^/ml, and recombinant Rluc-PEDF uptake was measured 24 h later. Rluc-PEDF (500 nM) was added to the supernatant of the medium and then the medium was replaced 2 h later. The cells were washed several times to ensure that no Rluc-PEDF remained in the supernatant, and subsequently, the reporter assay substrate was added (1:1000, P1112, Promega, Madison, WI). Then, fluorescence was detected at 488 nm in the bEnd.3 cells after 1 h.

### Assay of Recombinant Rluc-PEDF Crossing the Blood–Brain Barrier (BBB) *In Vitro*

Diluted fibronectin (FN) solution (2.1 μg/cm^2^) was placed on each filter insert (Transwell, polycarbonate membrane, 0.4 μm pore size, 6.5 mm diameter, 24 units/24-well plate, #3413, Costar, Corning, NY). The well plate was tilted several times to ensure that all inserts were covered with the FN solution. The well plate treated with FN solution was incubated at 37 °C for at least 30 min but no longer than 1 h. Excess FN solution was aspirated very carefully before seeding the cells. The function of FN is to promote cell adhesion and mimic cell basement membrane. The bEnd.3 cells (0.25 ml of 2 × 10^5^ cells/ml) were seeded into each chamber of the apical part (upper chamber) containing the filter membrane insert. Complete growth media (0.75 ml) was added to the lower chamber of each well. The cells were incubated for 7 days to ensure that the cells were tightly connected [36]. Rluc-PEDF was added to the medium of the upper chamber. 100 μl of medium was aspirated from the lower chambers 2 h later. Subsequently, the Renilla fluorescein substrate (1:1000) was added, and the fluorescence was detected at 488 nm at 1 h using a multifunctional microplate reader (SpectraMax M5, Molecular Devices, Sunnyvale, Silicon Valley). The fluorescence intensity was used to qualitatively reflect the Rluc-PEDF content.

### Assay of Recombinant Rluc-PEDF Crossing the BBB *In Vivo*

The SAMP8 mice were injected with recombinant Rluc-PEDF (4 mg/kg) through the tail vein, and 10 h later, the tissues were collected for immunofluorescence staining and other assays. The hippocampal tissue was homogenized. Then, substrate (1:1000) was added, and the Rluc-PEDF concentration was determined at 1 h using a multifunctional microplate reader (SpectraMax M5, Molecular Devices) with an excitation wavelength of 488 nm.

### RNA Isolation and Real-Time Quantitative PCR (RT-qPCR)

Cellular total RNA was extracted with TRIzol (Invitrogen, Waltham, MA) according to the manufacturer’s protocol. 500 ng of cDNA was used for RT-qPCR analysis using iQ SYBR Green Supermix and the iCycler Real-time PCR Detection System (Bio-Rad, Hercules, CA). Relative mRNA quantities were determined using the comparative cycle threshold (2^−∆∆Ct^) method. The sequences of PCR primers are shown in Supplementary Table 4. β-Actin was used for normalization.

### Western Blotting

Brain tissues and cells were lysed for total protein extraction using SDS buffer. Protein concentrations were determined using a Bio-Rad DC protein assay kit (Bio-Rad Laboratories) according to the manufacturer’s protocol. Equal amounts of protein were subjected to Western blotting analysis. Antibodies included those specific for Aβ (ab201060, Abcam, Cambridge Science Park, Cambridge, UK), PEDF (MAB1059, Millipore, Boston, MA), PS1 (ab76083, Abcam), phosphorylated c-Jun N-terminal kinase (P-JNK; #3270S, Cell Signaling Technology, Boston, MA), JNK (ab119944, Abcam), BACE1 (#5606S, Cell Signaling Technology), APP (#2452S, Cell Signaling Technology), low-density lipoprotein receptor-related protein 6 (LRP6; SC-25317, Santa Cruz, CA), phosphorylated extracellular signal-regulated kinase (P-ERK; SC-7383, Santa Cruz), ERK (SC-94, Santa Cruz), p38 mitogen-activated protein kinase (MAPK; #9211S, Cell Signaling Technology), P-p38 MAPK (#9212S, Cell Signaling Technology), and β-actin (A544, 2 ml, Sigma).

### ELISA

To quantify PEDF, the serum was centrifuged at 8000 rpm for 10 min. To quantify Aβ42 in brain tissue, 50 mg of mouse brain tissue homogenate was extracted using 2 ml of PBS with a protease inhibitor cocktail. The extracts were centrifuged at 12,000 × g for 30 min at 4 °C to remove insoluble materials. The supernatant fractions were analyzed by ELISA after total protein determination. To quantify Aβ42 produced from PC12 and APP-PS1(M146L) cells, the centrifuged cell supernatants were ultrafiltered by ultrafiltration tubes (4-kDa Millipore). There was a 5-fold increase in APP-PS1(M146L) and PC12 supernatants. Cell homogenates were extracted using 1 ml of PBS with a protease inhibitor cocktail. Protein concentrations were determined according to the manufacturer’s protocol. PEDF and Aβ42 in human serum, cell supernatants, or cell lysates were measured with a PEDF ELISA kit (DY1177-05, R&D Systems, MN) and a human Aβ42 ELISA kit (HKB3544, Life, MA). PEDF, Aβ42, β-secretase, and γ-secretase levels in mouse serum, cell supernatants, or cell lysates were measured with a mouse PEDF ELISA kit (PED613-Mouse, Life), a mouse Aβ42 ELISA kit (27721, IBL, Germany), a fluorometric β-secretase activity assay (ab65357, Abcam), and a mouse γ-secretase ELISA kit (CSB-E12142m, CUSABIO) according to the manufacturers’ instructions.

### Immunofluorescence

Brain tissues were removed after perfusion to flush away the blood and then fixed in ice-cold 4% paraformaldehyde overnight. Then, the tissues were dehydrated in a gradient of sucrose solutions (10%, 20%, 30%) overnight before tissue sectioning. For cell immunofluorescence, cells were plated on culture slides and then fixed in ice-cold 4% paraformaldehyde for 30 min. Then, the samples were prepared according to the protocol as described previously [26]. The slices were visualized under a confocal microscope (Axio Observer Z1, ZEISS, Jena, Germany). Antibodies included those specific for Aβ (ab201060, Abcam), PEDF (MAB1059, Millipore), PS1 (ab76083, Abcam), and Renilla luciferase (ab185926, Abcam).

### Statistical Analysis

The variability of the results is expressed as the SD (mean ± SD). For all studies comparing more than two groups, one-way ANOVA was used followed by the least significant difference *t* test for multiple comparisons among groups. Behavioral data from the training period were first assessed for normality and sphericity using Shapiro–Wilks and Mauchly’s tests, respectively, and then analyzed using repeated-measures ANOVA. Statistical significance was set at *p* < 0.05. All data were analyzed in SPSS (version 22, IBM, Armonk, NY).

## Results

### PEDF Levels Were Reduced in AD Patients and in the Mouse Model

**Fig. 1:**
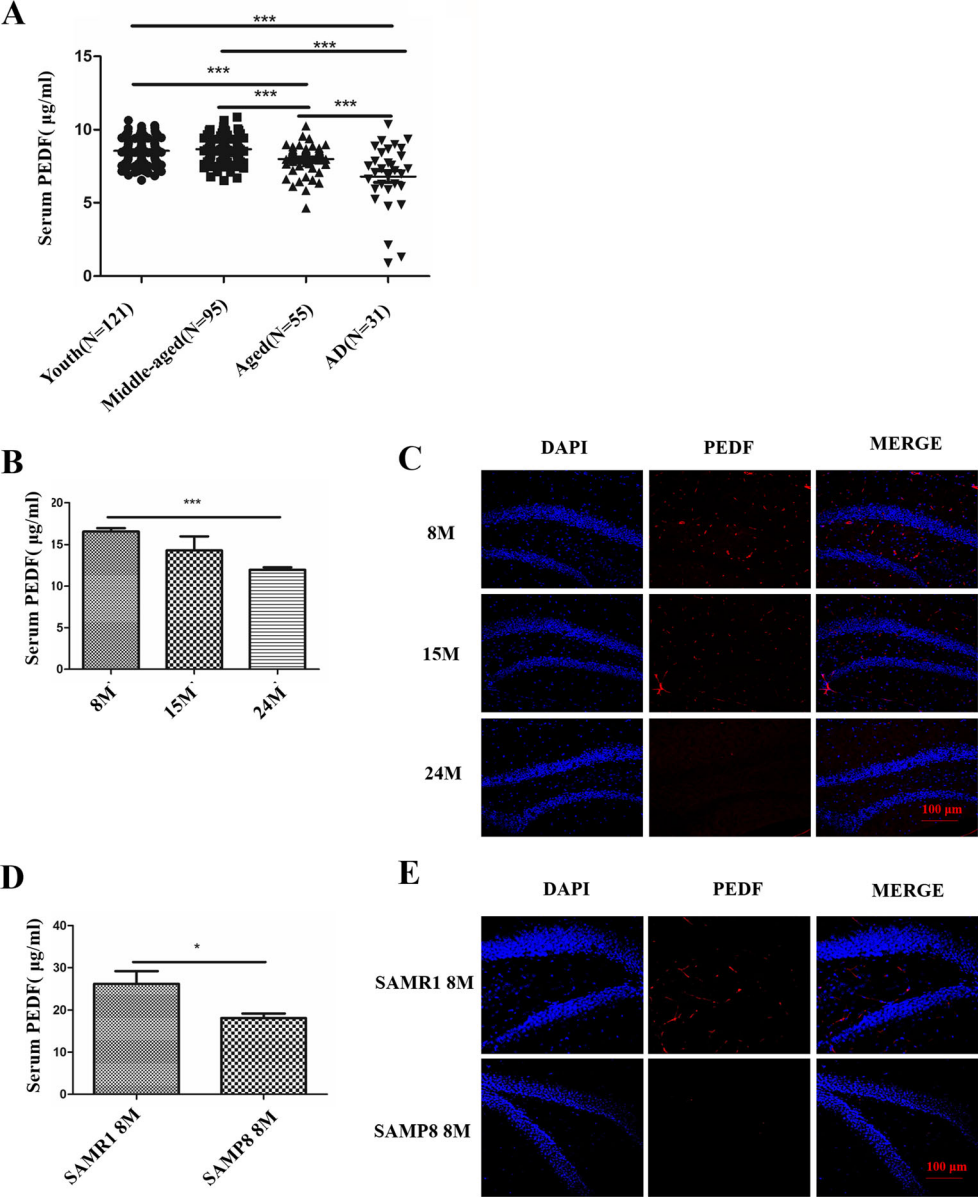
PEDF was reduced in AD patients and a mouse model of AD. (**A**) Serum PEDF content was quantified by ELISA in normal subjects and AD patient samples. The normal samples were divided into three groups by ages: the youth group (25–45 years; *N* = 121), the middle-aged group (46–60 years; *N* = 95), and the old-aged group (> 60 years; *N* = 55). The AD patients comprised one group (50–91 years; average = 70; *N* = 31). (**B**) Serum PEDF content was quantified by ELISA in C57BL/6 WT mouse samples. *N* = 6 in each group. (**C**) PEDF staining was performed in C57BL/6 WT mouse hippocampal tissue. Scale bar, 100 μm. (**D**) Serum PEDF content was quantified by ELISA in the SAMR1 and SAMP8 mice. *N* = 4 in each group. (**E**) PEDF staining was performed in the SAMR1 and SAMP8 mouse hippocampal tissue. Scale bar, 100 μm. Error bars represent the standard deviation (SD); one asterisk, *p* < 0.05; three asterisks, *p* < 0.001

To evaluate the relevance of PEDF on AD, we first tested serum samples of 31 AD patients (50–91 years old; average = 70 years old) and 271 normal controls (the youth group: 25–45 years old, *N* = 121; the middle-aged group: 46–60 years old, *N* = 95; and the old-aged group: > 60 years old, *N* = 55). Similar to previous reports [33, 37], the concentration of serum PEDF was significantly lower in the old-aged group (7.99 ± 0.13 μg/ml) than that in the youth (8.55 ± 0.0.8 μg/ml, *p* < 0.001) and middle-aged (8.67 ± 0.09 μg/ml, *p* < 0.001) groups. Interestingly, the PEDF level was significantly reduced in the AD group (6.80 ± 0.40 μg/ml) compared to that in the old-aged normal group (*p* < 0.05) and that in the other normal groups (*p* < 0.001; Fig. 1A). When examined in C57BL/6 wild-type (WT) mice, PEDF was reduced in both the serum samples and hippocampal tissue with aging (Fig. 1B, C). Furthermore, PEDF was reduced in the AD model SAMP8 mice compared with that in the control SAMR1 mice in serum and hippocampal tissue (Fig. 1D, E). Collectively, PEDF levels were decreased in AD patients and the mouse model, which suggested that PEDF might have an essential role in the AD process.

### Cognitive Impairment in the MWM in the SAMP8 Model Was Attenuated by PEDF

**Fig. 2:**
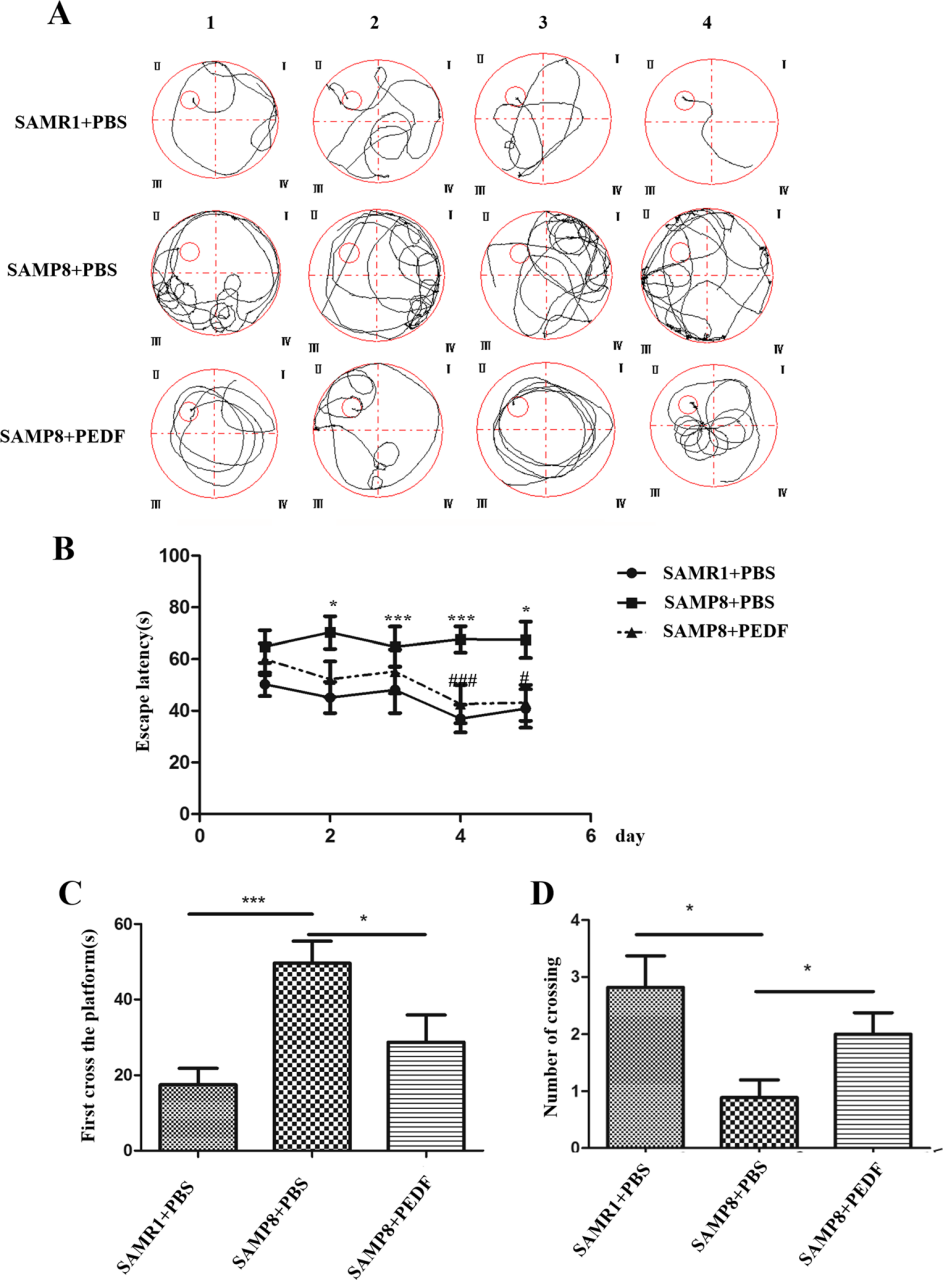
The cognitive impairment in SAMP8 mice was attenuated by PEDF, as assessed through the Morris water maze. Effect of treatment with PEDF-induced alterations in acquisition trials in the Morris water maze test: (**A**) the track diagram and (**B**) escape latency. One asterisk, *p* < 0.05; three asterisks, *p* < 0.001, *versus* the SAMR1 normal control group at the same time point; one number sign, *p* < 0.05; three number signs, *p* < 0.001, *versus* the SAMP8 untreated group at the same time point. Effect of PEDF treatment with on probe trials in the Morris water maze test: (**C**) time to reach the target region (that previously contained the platform) and (**D**) number of times crossing the target region. Error bars represent the standard deviation (SD); one asterisk, *p* < 0.05, three asterisks, *p* < 0.001

Although PEDF is a well-known neurotrophic factor, its effect on AD has not been documented. Of note, the escape latency to reach the platform was significantly increased in the untreated SAMP8 mice compared with that in the normal control SAMR1 mice, whereas the escape latency of the SAMP8 mice administered PEDF was significantly reduced compared to that in the untreated SAMP8 mice (Fig. 2A, B). The average swimming speeds were unchanged among these groups. The details of the average latency and average swimming speed of each group for 5 days are shown in Supplementary Table 2 and Supplementary Table 3. Furthermore, the time to reach the target region (that previously contained the platform) was increased in the SAMP8 mice (49.65 ± 5.81 s) in comparison with the time to reach the target in the SAMR1 mice (17.45 ± 4.35 s). However, the time required to reach the target region by the SAMP8 mice treated with PEDF (28.78 ± 7.19 s) was shorter and approached the time of the SAMR1 mice (Fig. 2C). Accordingly, the number of target region crossings was lower in the SAMP8 mice (0.89 ± 0.31) than that in the SAMR1 mice (2.82 ± 0.55). Nevertheless, the number of crossings was increased in the SAMP8 mice treated with PEDF (2.00 ± 0.38) compared with that in the untreated SAMP8 mice (0.89 ± 0.31; Fig. 2D). Taken together, these results indicated that PEDF could protect the AD model SAMP8 mice from cognitive impairment.

### Recombinant Rluc-PEDF Crossed the BBB *In Vitro* and *In Vivo*

**Fig. 3:**
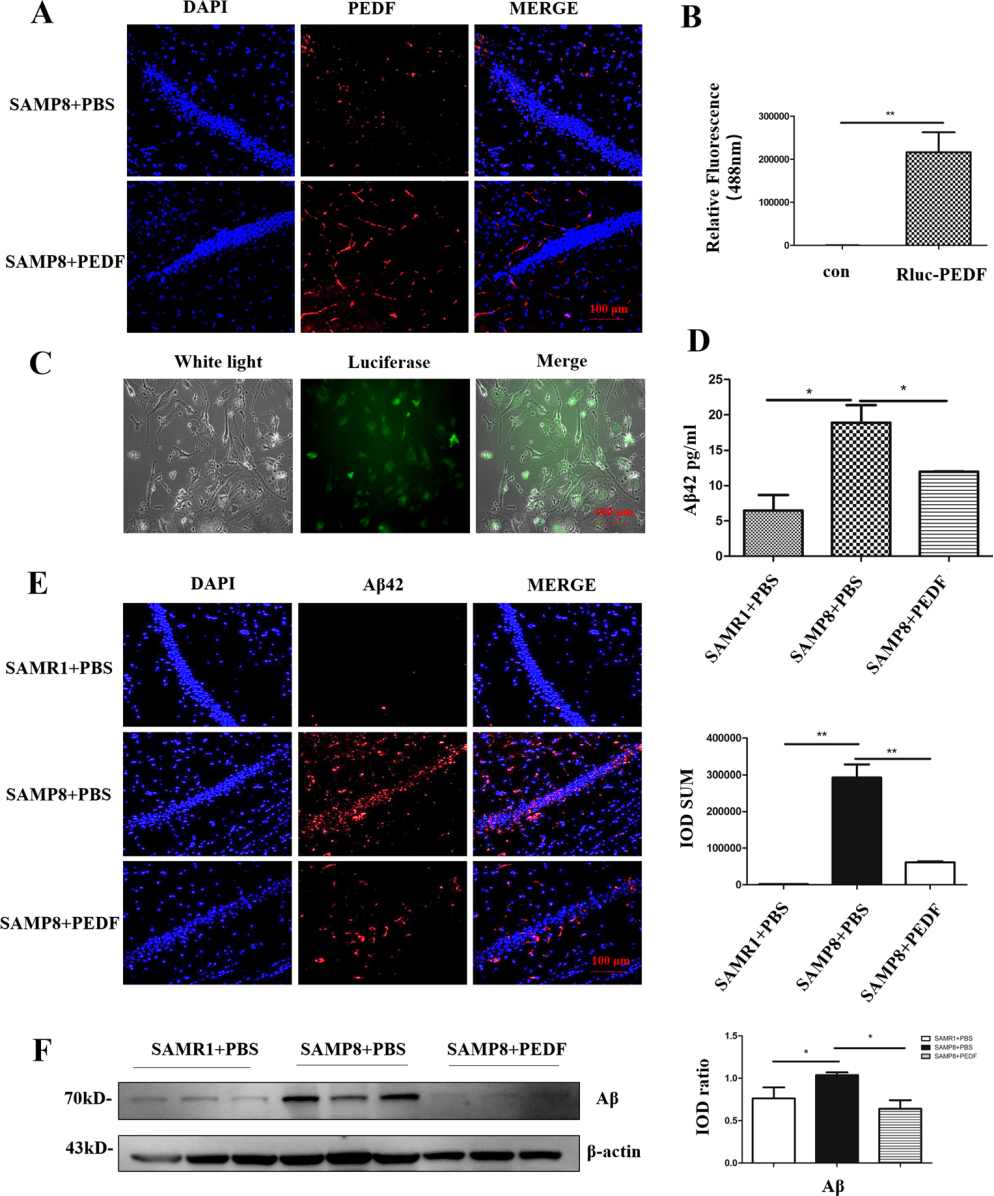
Increased Aβ42 in SAMP8 mice was attenuated with PEDF treatment. (**A**) PEDF staining was performed on hippocampal tissue after the Morris water maze. Scale bar, 100 μm. (**B**) Assay of recombinant Rluc-PEDF crossing the blood–brain barrier (BBB) *in vitro*. Detection of luciferase in the lower chamber using an excitation wavelength of 488 nm. (**C**) Assay of recombinant Rluc-PEDF uptake by bEnd3 *in vitro*. Detection of luciferase in bEnd.3 cells using an excitation wavelength of 488 nm. Scale bar, 100 μm. (**D**) ELISA analysis of Aβ42 expression in hippocampal tissue. (**E**) Aβ42 staining was performed on mouse hippocampal tissue. The staining was quantified using Image-Pro Plus (IPP) (*N* = 3). Scale bar, 100 μm. (**F**) Protein levels of Aβ were determined by Western blot in hippocampal tissue. β-Actin served as a loading control. The bands were quantified using ImageJ software, and the data were transformed and normalized relative to β-actin (*N* = 3). Error bars represent the standard deviation (SD); one asterisk, *p* < 0.05; two asterisks, *p* < 0.01

To determine whether PEDF could cross the BBB, we first examined the PEDF in the mouse hippocampal tissue after MWM test. Immunofluorescence data demonstrated that PEDF was increased in the SAMP8 mice treated with PEDF (Fig. 3A, S1B). In addition, in the assay of recombinant Rluc-PEDF crossing the BBB *in vitro*, we detected a strong fluorescent signal in the lower chamber 2 h after adding recombinant Rluc-PEDF in the upper chamber, suggesting that PEDF may cross the *in vitro* BBB model (Fig. 3B). Similarly, recombinant Rluc-PEDF was taken up by bEnd.3 cells (mouse brain microvascular endothelial cells) after 2 h of incubation (Fig. 3C). Furthermore, exogenous recombinant Rluc-PEDF was present in the hippocampus of the SAMP8 mice (Fig. S1C, S1D) and other tissues such as the kidney and liver (Fig. S2A) at 10 h after tail vein injection. By adding the substrate Renilla luciferase to the hippocampal tissue homogenate, a strong fluorescent signal was detected compared to that in the untreated group (Fig. S1B), suggesting that PEDF may cross the BBB *in vivo* as well.

### Increased Aβ42 in the SAMP8 Model Was Attenuated by PEDF Administration

We detected Aβ42 expression in the experimental mouse hippocampal tissue, and high levels of Aβ42 were corroborated in the SAMP8 mice (18.89 ± 2.48 pg/ml) compared with those in the SAMR1 mice (6.47 ± 2.21 pg/ml). Moreover, Aβ42 expression was downregulated in the SAMP8 mice with the administration of PEDF (11.97 ± 0.06 pg/ml; Figs. 3D, E, S2B). Consistent with this result, Western blot analysis showed that Aβ oligomer expression was extraordinarily increased in the SAMP8 mice compared to that in the SAMR1 mice, whereas the administration of PEDF significantly reduced the expression of Aβ oligomers in the SAMP8 mice (Fig. 3F). Therefore, we concluded that the cognitive impairment in the SAMP8 model was likely attenuated by PEDF-induced downregulation of Aβ42 in the hippocampus.

### Aβ42 Was Upregulated in PEDF KO Mice

**Fig. 4:**
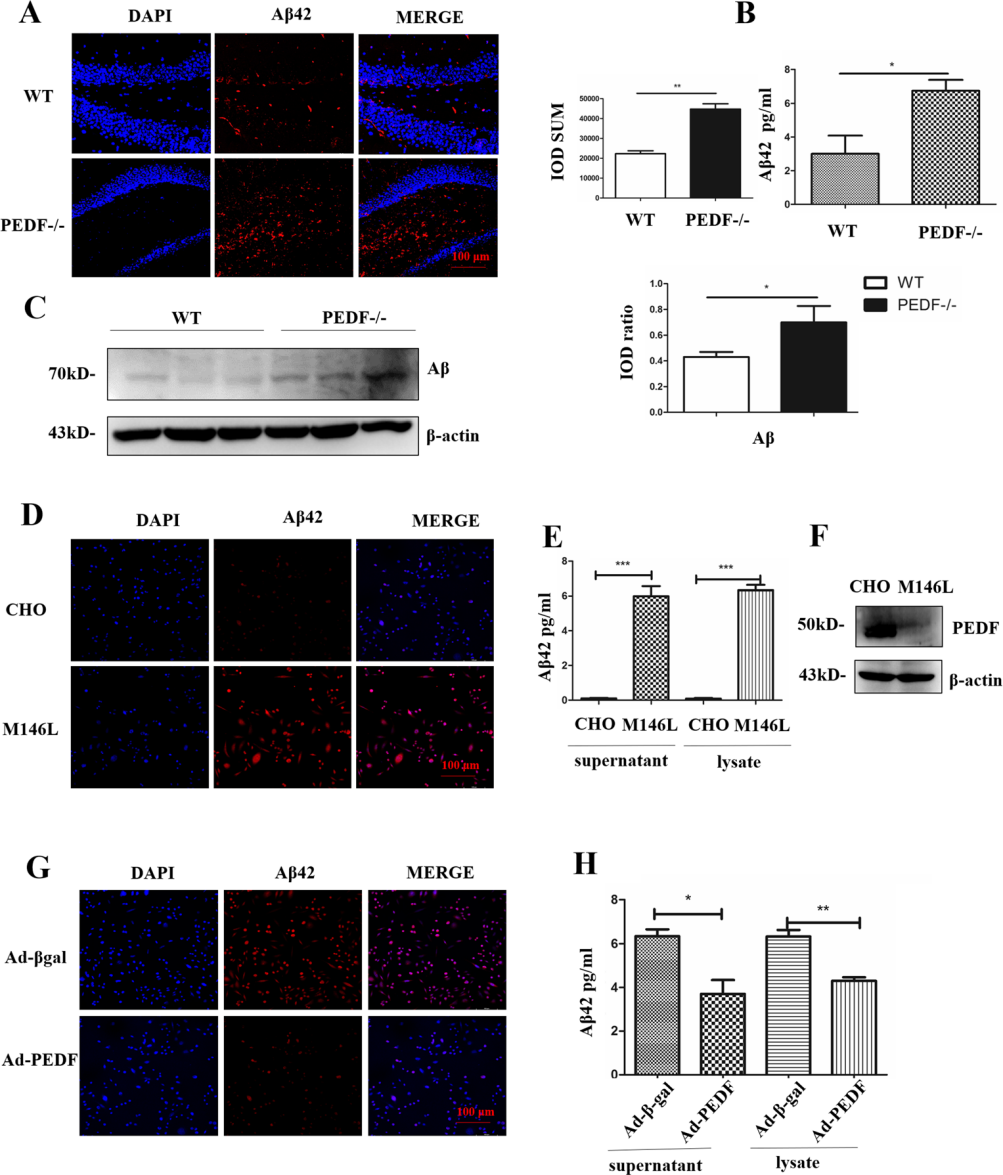
The expression of Aβ42 was regulated by PEDF. (**A**) Aβ42 staining was performed in WT and PEDF KO hippocampal tissue. The staining was quantified using Image-Pro Plus (IPP) (*N* = 3). Scale bar, 100 μm. (**B**) ELISA analysis of Aβ42 expression in WT and PEDF KO hippocampal tissue. β-Actin served as a loading control. (**C**) Western blot analysis of Aβ expression in WT and PEDF KO hippocampal tissue. The bands were quantified using ImageJ software, and the data were transformed and normalized relative to β-actin (*N* = 3). (**D**) Aβ42 staining was performed in CHO and APP-PS1(M146L) cells. Scale bar, 100 μm. (**E**) ELISA analysis of Aβ42 expression in cell supernatants and lysates. (**F**) Protein levels of PEDF were determined by Western blot in cells. β-Actin served as a loading control. (**G**) Aβ42 staining was performed on APP-PS1(M146L) cells 72 h after transfection of adenovirus. Scale bar, 100 μm. (**H**) ELISA analysis of Aβ42 expression in cell supernatants and lysates 72 h after transfection of adenovirus. Error bars represent the standard deviation (SD); one asterisk, *p* < 0.05; two asterisks, *p* < 0.01, three asterisks, *p* < 0.001

To identify the connection between Aβ42 and PEDF, we investigated Aβ42 expression in PEDF KO mice (8 months old). As shown in Fig. 4A, B, the level of Aβ42 was significantly higher in PEDF KO (6.75 ± 0.63 pg/ml) mouse hippocampal tissue than that in the WT control tissue (3.00 ± 1.08 pg/ml). Consistently, Aβ oligomers were also increased in the PEDF KO mouse hippocampal tissue (Fig. 4C).

### Aβ42 Was Negatively Regulated by PEDF

APP-PS1(M146L) is a CHO cell line transfected with human APP-PS1 double genes [38]; these cells highly express Aβ42 both in the cell supernatant (5.99 ± 0.57 pg/ml) and lysate (6.33 ± 0.30 pg/ml) when compared with the untransfected CHO cells (supernatant, 0.10 ± 0.03 pg/ml; lysate, 0.09 ± 0.04 pg/ml; Fig. 4D, E). The expression of PEDF was lower in the APP-PS1(M146L) cells than that in the CHO cells (Fig. 4F). Aβ42 was further decreased in the APP-PS1(M146L) cells both in the supernatant (3.70 ± 0.64 pg/ml) and lysate (4.30 ± 0.16 pg/ml) after overexpressing PEDF for 72 h when compared with the control cells (supernatant, 6.34 ± 0.32 pg/ml; lysate, 6.33 ± 0.30 pg/ml; Fig. 4G, H). Moreover, Aβ42 was upregulated by the stable interference of PEDF but was downregulated by overexpressing of PEDF (Fig. S3A, S3B). These results suggested that the expression of Aβ42 was negatively regulated by PEDF.

### PEDF Negatively Regulated Aβ Generation by Inhibiting γ-Secretase Rather Than β-Secretase

**Fig. 5:**
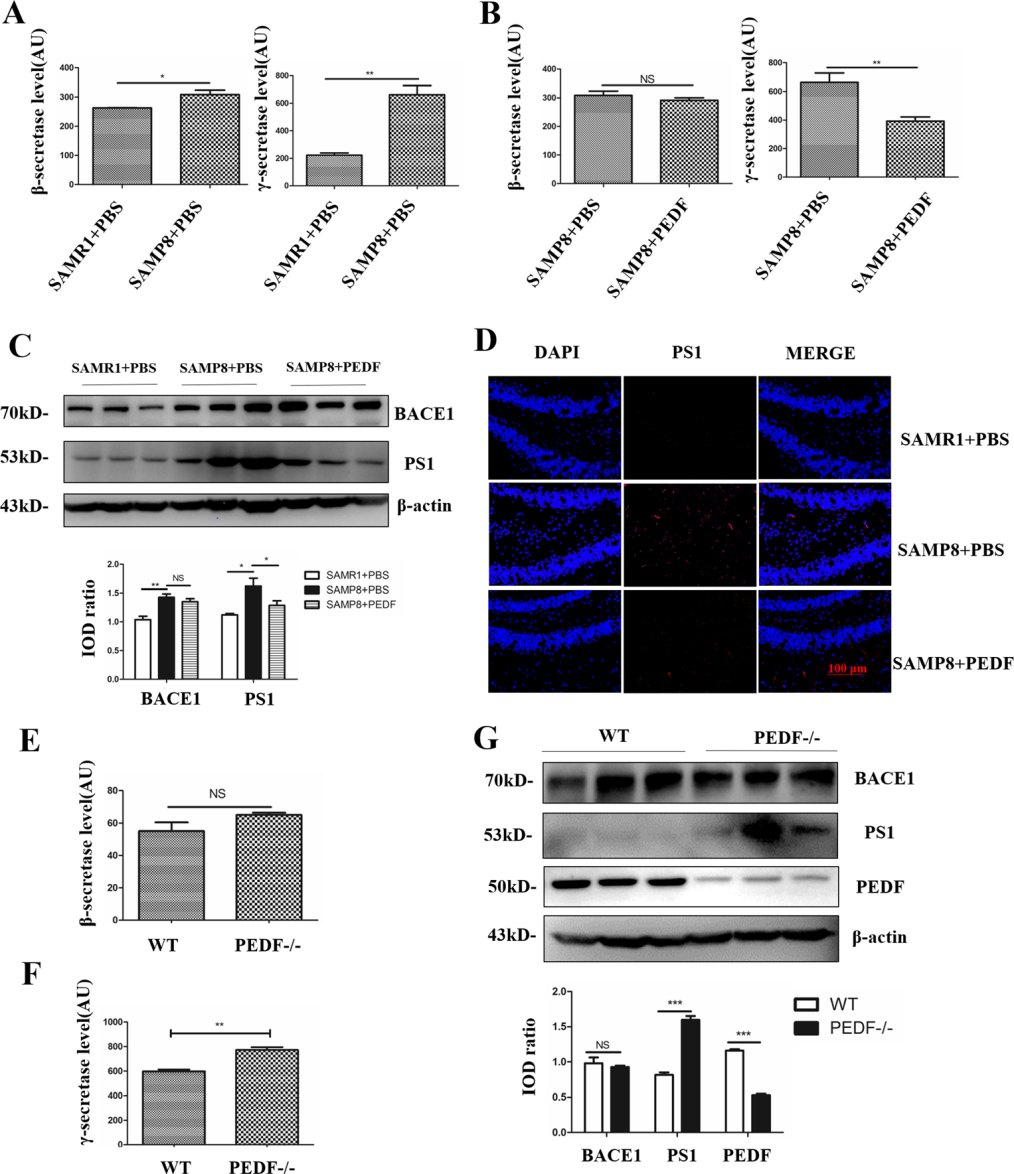
PEDF regulated Aβ production through γ-secretase, not β-secretase. (**A**) γ-Secretase activity was analyzed in mouse hippocampal tissue. (**B**) β-Secretase activity was analyzed in mouse hippocampus tissue. (**C**) Protein levels were determined by Western blot in mouse hippocampal tissue. β-Actin served as the loading control. The bands were quantified using ImageJ software, and the data were transformed and normalized relative to β-actin (*N* = 3). (**D**) Immunofluorescence staining of PS1 on mouse hippocampal tissue. Scale bar, 100 μm. (**E**) γ-Secretase activity was analyzed in 8-month-old WT and PEDF KO mouse hippocampal tissue. (**F**) β-Secretase activity was analyzed in 8-month-old WT and PEDF KO mouse hippocampal tissue. (**G**) Protein levels were determined by Western blot in 8-month-old WT and PEDF KO hippocampal tissue. β-Actin served as the loading control. The bands were quantified using ImageJ software, and the data were transformed and normalized relative to β-actin (*N* = 3). Error bars represent the standard deviation (SD); one asterisk, *p* < 0.05; two asterisks, *p* < 0.01, three asterisks, *p* < 0.001

Aβ originates from APP, which must undergo two sequential endoproteolytic steps of proteolysis by two crucial enzymes known as γ-secretase and β-secretase [2, 39]. The activity levels of these two enzymes were increased in the SAMP8 mice (Fig. 5A) [40]. In addition, PEDF treatment potently inhibited γ-secretase expression, whereas little effect was observed for β-secretase expression (Fig. 5B). Furthermore, increased PS1 expression in the SAMP8 mice was attenuated with the administration of PEDF (Fig. 5C, D). Consistent with this finding, γ-secretase activity was increased in PEDF KO mice, while there was no difference in β-secretase activity (Fig. 5E, F). Moreover, a high level of PS1 expression was detected in PEDF KO mice (Fig. 5G), suggesting that PEDF regulates Aβ production by inhibiting PS1 expression, resulting in γ-secretase dysfunction.

### PEDF Downregulated the Expression of PS1 Through the MAPK/JNK Pathway

**Fig. 6:**
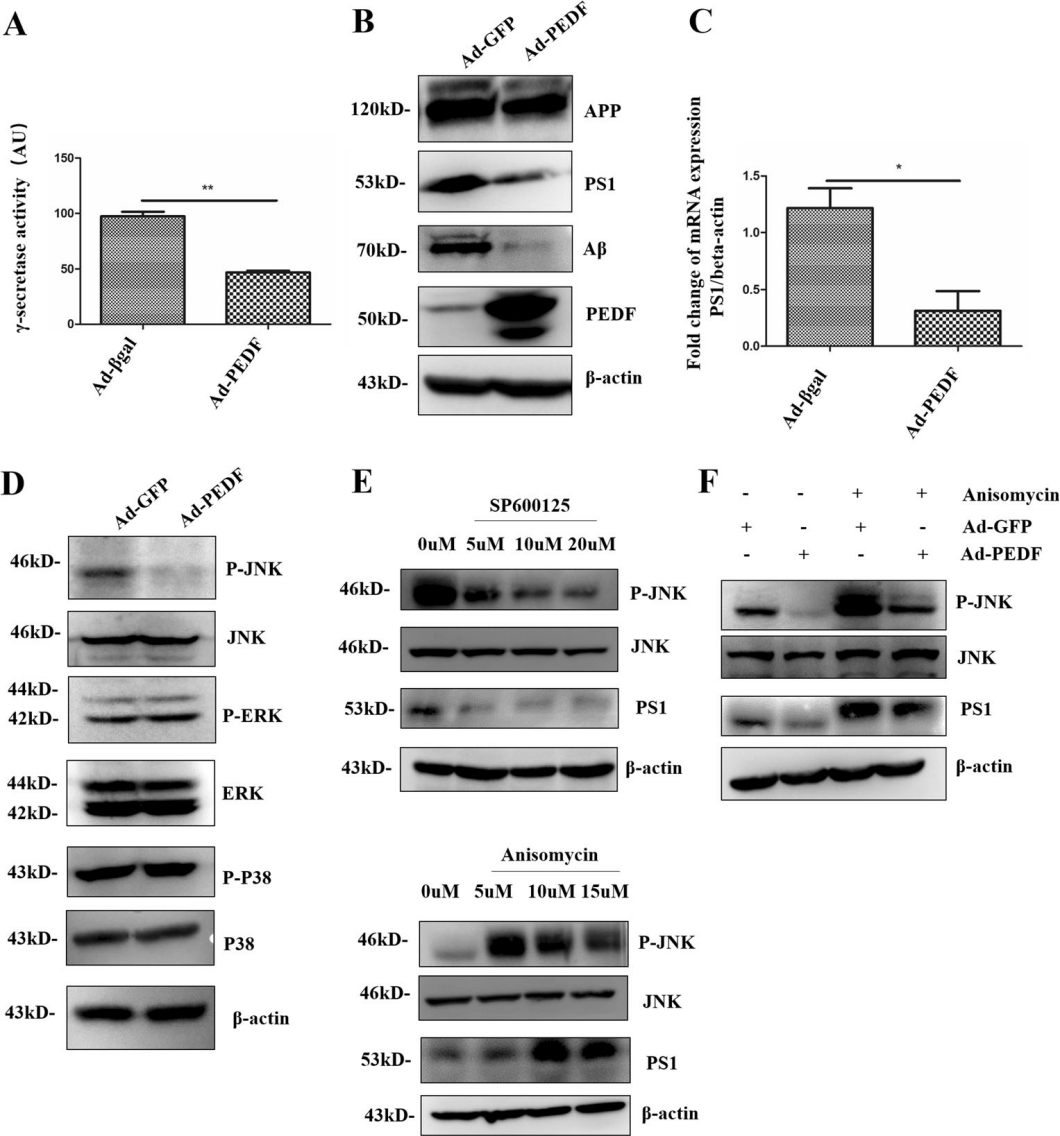
PEDF regulated the expression of PS1 through the JNK pathway. (**A**) APP-PS1(M146L) cells were infected with viruses expressing PEDF for 72 h followed by γ-secretase activity analysis. Ad-βgal served as a control. (**B**) Protein levels were determined by Western blot after APP-PS1(M146L) cells were infected with viruses expressing PEDF for 72 h. Ad-GFP served as control. (**C**) APP-PS1(M146L) cells were infected with viruses for 72 h followed by RT-PCR analysis of PS1. β-Actin served as an internal reference. (**D**) Western blot analysis of the key enzyme protein levels in the MAPK pathway. (**E**) APP-PS1(M146L) cells were treated with a JNK inhibitor or agonist for 2 h followed by Western blot analysis of protein levels. (**F**) Protein levels were determined by Western blot. APP-PS1(M146L) cells were infected with viruses expressing PEDF for 72 h followed by treatment with or without a JNK agonist for 2 h. Error bars represent the standard deviation (SD); one asterisk, *p* < 0.05; two asterisks, *p* < 0.01

**Fig. 7:**
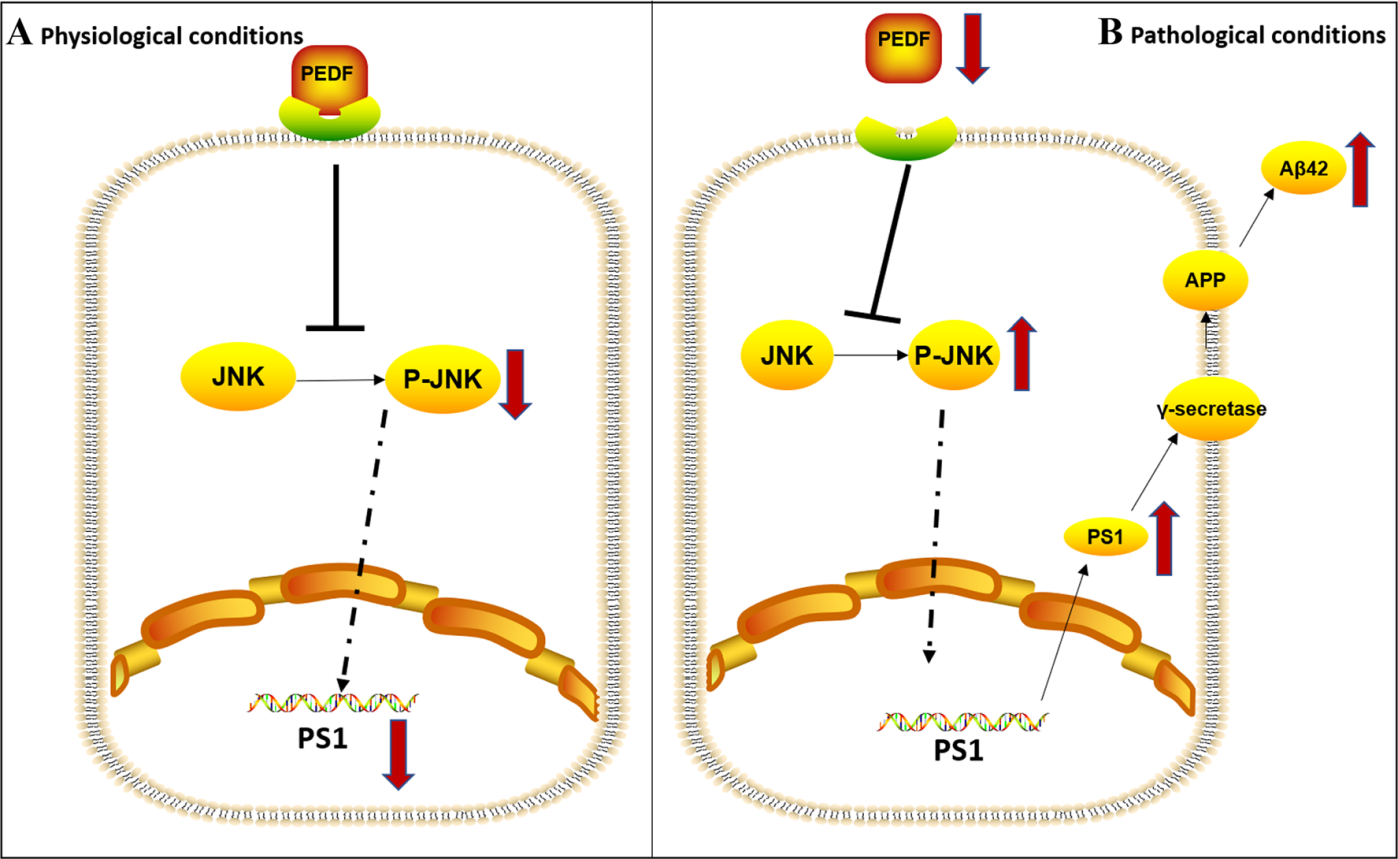
Simplified model depicting the pathway of Aβ42 regulated by PEDF. (**a**) The phosphorylation of JNK is inhibited by high levels of PEDF in the physiological state. (**b**) Aβ42 is upregulated by JNK pathway activation lack of PEDF in the pathological state

PS1 expression and γ-secretase activity were potently inhibited in APP-PS1(M146L) cells overexpressing PEDF (Fig. 6A, B). The results in the PC12 cells were consistent with those in the APP-PS1(M146L) cells (Fig. S3C). We further found that the translation of PS1 mRNA was also regulated by PEDF (Fig. 6C). The PS1 expression is controlled by the ERK and JNK pathway [40, 41]. Thus, we overexpressed PEDF in APP-PS1(M146L) cells to analyze MAPK variation. Notably, the phosphorylation of JNK was exclusively suppressed (Fig. 6D). Dramatically increased PS1 expression was observed following JNK activation whereas PS1 was decreased with attenuated JNK phosphorylation in APP-PS1(M146L) cells at 2 h after treatment with a JNK inhibitor or agonist, respectively (Fig. 6E). Dramatically increased PS1 by administration of JNK agonist was attenuated by treatment with PEDF (Fig. 6F). Phosphorylation of JNK and PS1 expression were also suppressed by PEDF protein treatment (Fig. 7, Fig. S3D). Taken together, these results suggested that the effect of PEDF on decreasing the expression of PS1, at least in part, occurred through the blockade of the JNK pathway.

## Discussion

Aβ42 aggregation precedes SP formation in neuronal damage, which eventually causes AD [2, 5]. The molecular basis underlying the pathogenesis of Aβ42 aggregation is not completely understood. The current study first demonstrated that serum PEDF levels were significantly decreased in AD patients. Moreover, serum and hippocampal PEDF expression levels were reduced and were associated with an increased Aβ42 level in SAMP8 mice. Administration of recombinant PEDF ameliorated memory impairment in SAMP8 mice. PEDF downregulated the expression of Aβ42 in PEDF KO mice and SAMP8 mice through inhibiting γ-secretase activity. Furthermore, our studies revealed that PEDF could downregulate PS1 expression via the JNK pathway, which subsequently suppressed Aβ42 expression. These findings are the first to establish that a deficiency in PEDF levels in serum and brain is associated with AD and may provide a novel theoretical basis and intervention targets for AD.

PEDF is a multifunctional protein characterized by its broad functions, including anti-inflammation [42], antiangiogenesis [43], antitumorigenesis [27], and antimetastasis [26, 44] activities. Previous studies including ours have demonstrated that PEDF can be expressed in almost all tissues and different cell types containing liver, adipose, epithelial cells, endothelial cells, neurons, etc. [16, 45–48]. The liver is regarded as the main source of PEDF [48–50]. Adipose tissue is another major organ that secretes PEDF [35, 46]. Therefore, serum PEDF may be sourced from all of these tissues, as PEDF is a secreted protein [51]. Considering a previous report showing that the concentration of PEDF in CSF is consistent with that in serum [18], we speculate that systemic PEDF may influence the content of PEDF in the CNS and that the decrease in PEDF expression with aging and to a greater extent in AD should be systemic.

Similar to previous reports [33, 37], our results show that the concentration of serum PEDF was lower in the old-aged group than those in young and middle-aged groups (Fig. 1A); correspondingly, reduced serum and hippocampal PEDF levels were observed in aged C57BL/6 mice (Fig. 1B, C). Importantly, different from previous reports, our study demonstrated that endogenous PEDF was further reduced in AD patients compared with age-matched controls (Fig. 1A). This decrease was confirmed in SAMP8 mice (Fig. 1D, E). The changes in PEDF content in AD are controversial. Previous studies showed PEDF was elevated [18, 19] or unchanged [20, 21] in AD. Our data showed serum PEDF was decreased in AD consistent with a previous study that PEDF was also decreased in patients with frontotemporal dementia, another neurodegenerative disease [52]. Our report differed in age range from those of other studies. The age of AD patients and normal controls was matched in our study (average age: AD = 69.65 ± 1.65 years, control = 73.00 ± 1.22 years). However, other studies showed a large range of age, especially in the group of normal controls (average age: AD = 70.750 ± 7.424 years, control = 51.632 ± 21.798 years [18]; AD = 73 ± 7 years, control = 56 ± 17 years [20]). The difference may also be attributable to the number of patients. The numbers of patients were relatively low in the previous studies {AD (*N* = 12), control (*N* = 19) [18]; AD (*N* = 5), control (*N* = 3) [19]; AD (*N* = 27), control (*N* = 27) [20]}. Our study tested more AD and normal samples {AD (*N* = 55), control (*N* = 31)}. We tested PEDF content using an ELISA kit, which was more accurate than immunohistochemistry methods [19]. Some other factors, such as metabolic conditions, could also affect PEDF levels. We and others have shown that metabolic phenotype affects PEDF levels [34, 53]. In the present study, we excluded samples from patients with abnormal metabolic conditions. It is not clear whether other studies excluded this factor.

Overall, many factors may influence PEDF levels in aging and in AD patients, resulting in conflicting outcomes. The present study may be more credible as our design considered more influencing factors, including age range, patient number, and quantitation methods, and excluded metabolic abnormalities.

Overexpressing human PEDF in rat brains can attenuate ischemic brain damage in a rat middle cerebral artery occlusion (MCAO) model [17]. Aβ42 aggregation is a major pathologic and early feature of AD [2]. We found that Aβ42 highly expressed in PEDF KO mice appeared from 4 months old (data not shown) and significant difference occurred at 8 months old (Fig. 4A–C), suggesting that PEDF deficiency occurred before Aβ42 accumulated abnormally. Thus, PEDF could function as an important factor in AD pathogenesis and serve as a candidate target for treatment.

Currently, no effective disease-modifying treatment is available for AD, although the US Food and Drug Administration has approved several drugs to treat this disease [54]. The BBB limits the uptake of most drugs, leading drug development into a bottleneck [55]. It is generally assumed that small-molecule drugs may cross the BBB, yet only ~ 2% of all small-molecule drugs are brain penetrating, and even fewer of these drugs are active in the central nervous system (CNS) [55, 56]. The carrier-mediated transport (CMT) systems are members of the solute carrier (SLC) gene families, responsible for BBB nutrient transport, including glucose transporter 1 (GLUT1), monocarboxylate transporter 1 (MCT1), large neutral amino acid transporter (LAT1), etc. [55]. Of note, here we showed that recombinant PEDF could cross the BBB *in vitro* (Fig. 3B, C) and *in vivo* (Figs. 3A, S1C, S1D). Because of the physiological differences between the human and mouse BBB [57], the efficiency of PEDF transport through the BBB in clinical samples needs to be further explored. If effective, intravenous injection of PEDF would be safer for AD patients.

In this study, we chose SAMP8 mice as the animal model of AD because nontransgenic SAMP8 mice have the advantage of representing late-onset, age-related sporadic AD (SAD), which up to 95% of AD patients are diagnosed with [58]. Similar to other studies [8, 59], both BACE1 and PS1 levels were increased in SAMP8 mice (Fig. 5C). With the administration of recombinant PEDF, PS1 expression was negatively regulated, while there was no change in the expression of BACE1 (Fig. 5C). PS1 levels and activity were also increased in PEDF KO mice (Fig. 5G), suggesting that PEDF regulated Aβ42 via PS1 rather than BACE1. JNK, known as a stress-activated protein kinase, is a subclass of the MAPK signaling pathway in mammalian cells. JNK regulates various processes in AD such as brain development and repair, neuroinflammation, neuronal death, and memory formation [60, 61]. Furthermore, impaired insulin resistance (IR) signaling has been reported during aging and in the AD brain [62], while prolonged activation of JNK contributes to IR desensitization [63]. JNK has been demonstrated to positively regulate the expression of BACE1 and PS1 [64]. Studies have shown that PS1 expression is regulated by ERK, as well as subfamilies of MAPK [40, 41]. Our results showed that JNK phosphorylation rather than ERK phosphorylation was affected by PEDF (Fig. 6D), suggesting that PEDF negatively regulates PS1 expression through inhibiting the JNK pathway.

Previous studies have revealed that decreased low-density lipoprotein receptor-related protein (LRP) expression is associated with positive staining of vessels for Aβ [65, 66]. In addition, deficiency in LRP6, regarded as the PEDF receptor [34], contributes to AD [67]. Using an LRP6 neutralizing antibody, the effect of PEDF on suppressing JNK phosphorylation was blocked (Fig. S3E), indicating that PEDF might regulate AD through LRP6. Whether LRP6 serves as a receptor of PEDF to mediate the downstream signaling pathway needs to be further investigated.

In summary, we confirmed that PEDF concentrations were reduced in AD patients. In addition, our study demonstrated for the first time that PEDF negatively regulates Aβ42 and that PEDF deficiency with aging might play a crucial role in the pathogenesis of AD. These findings might provide new insight into therapies for AD.

## Electronic supplementary material

Fig. S1 (A) The Renilla luciferase gene was inserted into a His-PEDF plasmid. The recombinant Rluc-PEDF constructed plasmid map is shown. (B) Immunofluorescence staining of PEDF in mouse hippocampal tissue. (C) Assay of recombinant Rluc-PEDF crossing BBB *in vivo*. Immunofluorescence staining of Renilla luciferase in mouse hippocampal tissue. (D) Assay of recombinant Rluc-PEDF crossing BBB *in vivo*. Add the substrate of Renilla luciferase followed by detection of luciferase in SAMP8 and SAMR1 mouse hippocampal tissue homogenate using an excitation wavelength of 488 nm. Scale bar, 100 μm. Error bars represent the standard deviation (SD); ^*^p < 0.05, ^**^p < 0.01, ^***^p < 0.001. (GIF 72 kb)

High Resolution Image (TIF 19812 kb)

Fig. S2 (A) Immunofluorescence staining of Renilla luciferase in mouse kidney and liver tissues. (B) Immunofluorescence staining of Aβ42 in mouse hippocampal tissue. The staining was quantified using Image-Pro Plus (IPP) (*N* = 3). Scale bar, 100 μm. (GIF 35 kb)

High Resolution Image (TIF 18638 kb)

Fig. S3 (A) ELISA analysis of Aβ42 expression in PC12 cell supernatants and lysates. (B) Western blot analysis of PEDF in PC12 cells with stable interference or overexpression of PEDF. (C) γ-secretase activity was analyzed in PC12 cells with stable interference or overexpression of PEDF. (D) Western blot analysis of protein levels in APP-PS1(M146L) cells 24 h after by administration of recombinant PEDF. (E) Protein levels were determined by Western blot after APP-PS1(M146L) cells were infected with viruses expressing PEDF for 48 h followed by application of an LRP6 neutralizing antibody for 24 h. Ad-GFP served as a control. (GIF 16 kb)

High Resolution Image (TIF 14780 kb)

ESM 4 (PDF 1224 kb)

ESM 5 (PDF 1224 kb)

ESM 6 (PDF 1224 kb)

ESM 7 (PDF 1224 kb)

ESM 8 (PDF 1224 kb)

ESM 9 (PDF 1224 kb)

ESM 10 (PDF 1224 kb)

ESM 11 (PDF 1224 kb)

ESM 12 (PDF 1224 kb)

ESM 13 (PDF 1224 kb)

ESM 14 (PDF 1224 kb)

ESM 15 (PDF 1224 kb)

ESM 16 (PDF 1224 kb)

ESM 17 (PDF 1224 kb)

ESM 18 (PDF 1224 kb)

ESM 19 (DOCX 21.7 kb)

ESM 20 (DOCX 17.9 kb)

ESM 21 (DOCX 13.2 kb)

ESM 22 (DOCX 12.5 kb)
