# Supplementary figures and images for: Pigment Epithelium-Derived Factor Plays a Role in Alzheimer’s Disease by Negatively Regulating Aβ42
Source: Neurotherapeutics. 2018 May 7;15(3):728–41. doi: 10.1007/s13311-018-0628-1 (PMC6095778; doi:10.1007/s13311-018-0628-1)

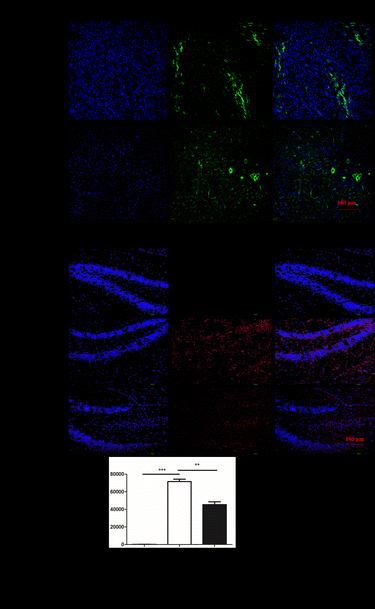

Supplement: Supplementary file 1 — (A) The Renilla luciferase gene was inserted into a His-PEDF plasmid. The recombinant Rluc-PEDF constructed plasmid map is shown. (B) Immunofluorescence staining of PEDF in mouse hippocampal tissue. (C) Assay of recombinant Rluc-PEDF crossing BBB in vivo. Immunofluorescence staining of Renilla luciferase in mouse hippocampal tissue. (D) Assay of recombinant Rluc-PEDF crossing BBB in vivo. Add the substrate of Renilla luciferase followed by detection of luciferase in SAMP8 and SAMR1 mouse hippocampal tissue homogenate using an excitation wavelength of 488 nm. Scale bar, 100 μm. Error bars represent the standard deviation (SD); *p < 0.05, **p < 0.01, ***p < 0.001. (GIF 72 kb) [file 13311_2018_628_Fig9_ESM.gif]

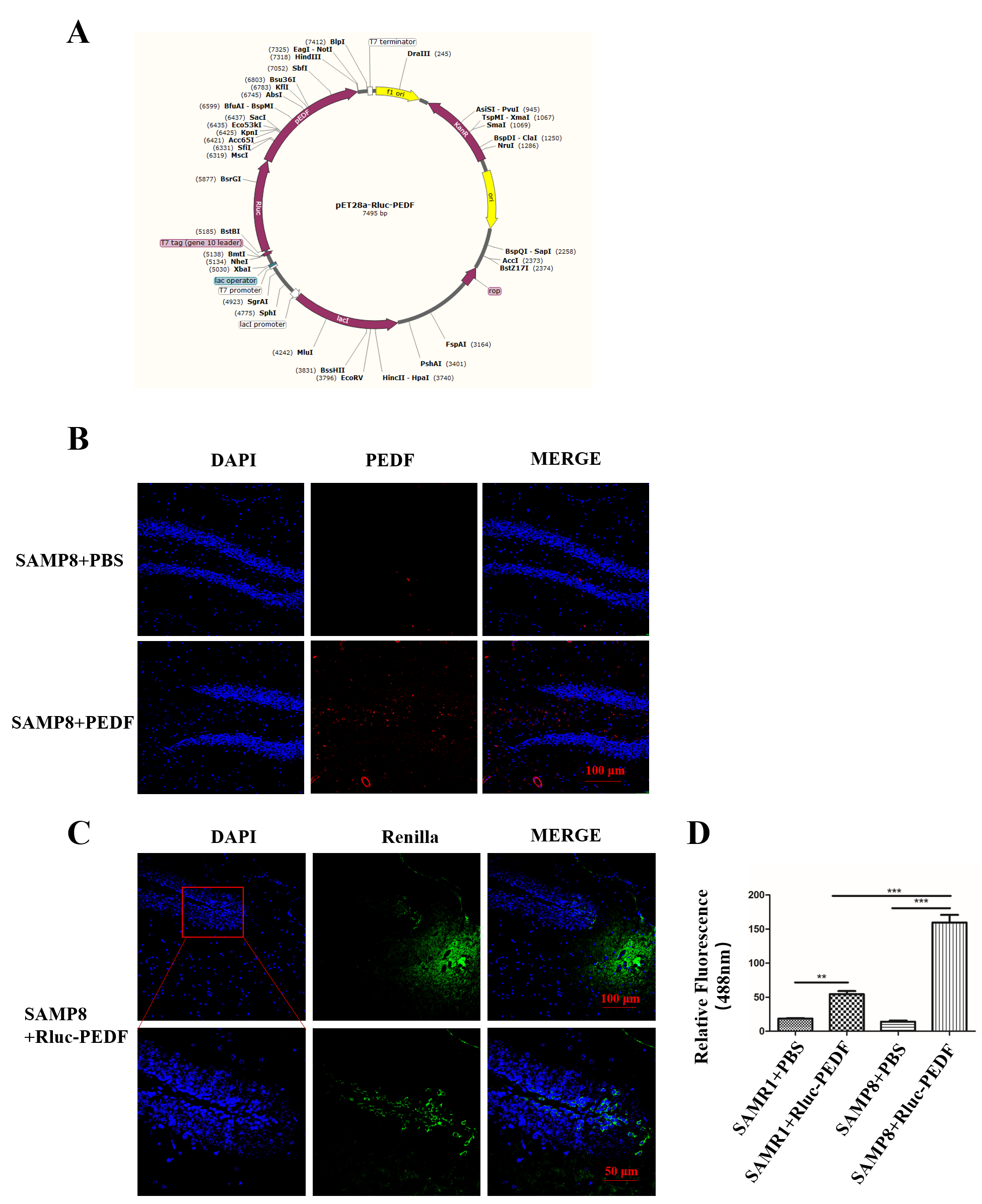

Supplement: Supplementary file 2 — High Resolution Image (TIF 19812 kb) [file 13311_2018_628_MOESM1_ESM.tif]

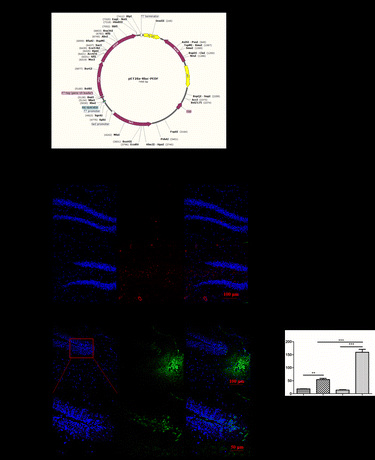

Supplement: Supplementary file 3 — (A) Immunofluorescence staining of Renilla luciferase in mouse kidney and liver tissues. (B) Immunofluorescence staining of Aβ42 in mouse hippocampal tissue. The staining was quantified using Image-Pro Plus (IPP) (N = 3). Scale bar, 100 μm. (GIF 35 kb) [file 13311_2018_628_Fig8_ESM.gif]

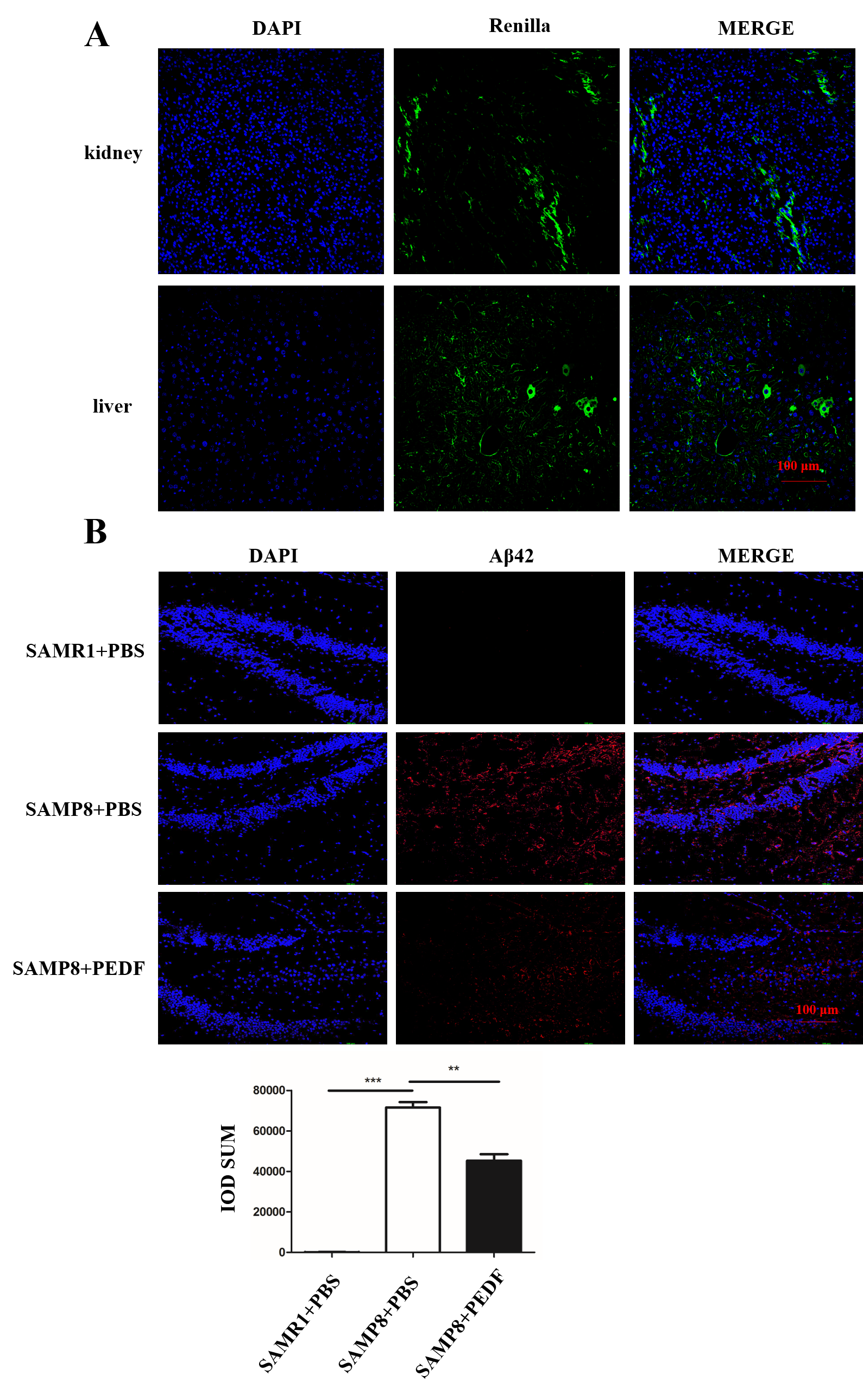

Supplement: Supplementary file 4 — High Resolution Image (TIF 18638 kb) [file 13311_2018_628_MOESM2_ESM.tif]

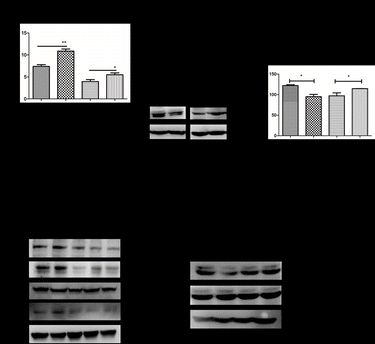

Supplement: Supplementary file 5 — (A) ELISA analysis of Aβ42 expression in PC12 cell supernatants and lysates. (B) Western blot analysis of PEDF in PC12 cells with stable interference or overexpression of PEDF. (C) γ-secretase activity was analyzed in PC12 cells with stable interference or overexpression of PEDF. (D) Western blot analysis of protein levels in APP-PS1(M146L) cells 24 h after by administration of recombinant PEDF. (E) Protein levels were determined by Western blot after APP-PS1(M146L) cells were infected with viruses expressing PEDF for 48 h followed by application of an LRP6 neutralizing antibody for 24 h. Ad-GFP served as a control. (GIF 16 kb) [file 13311_2018_628_Fig10_ESM.gif]

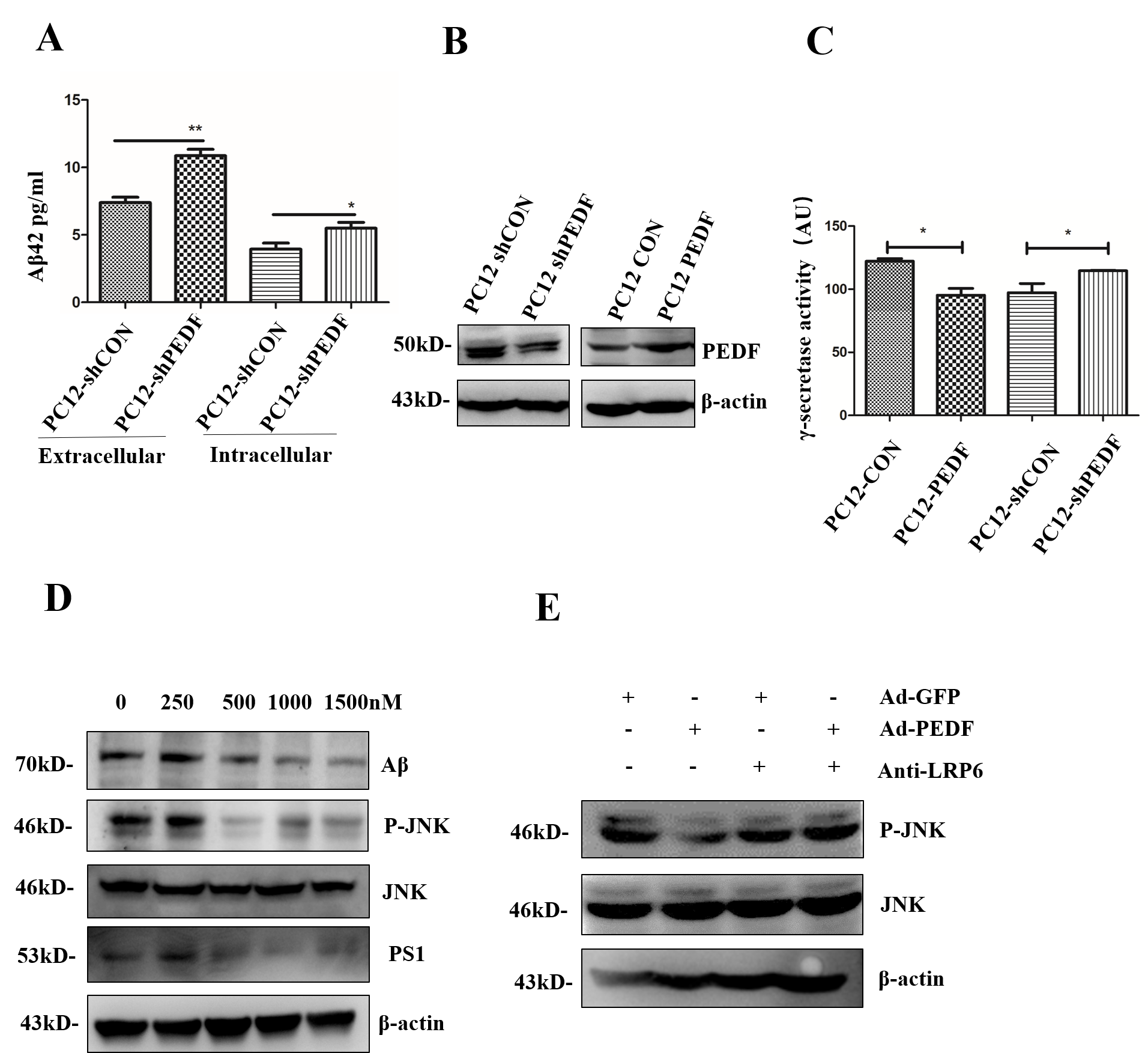

Supplement: Supplementary file 6 — High Resolution Image (TIF 14780 kb) [file 13311_2018_628_MOESM3_ESM.tif]
