# Supplementary material for: Pigment Epithelium-Derived Factor Plays a Role in Alzheimer’s Disease by Negatively Regulating Aβ42
Source: Neurotherapeutics. 2018 May 7;15(3):728–41. doi: 10.1007/s13311-018-0628-1 (PMC6095778; doi:10.1007/s13311-018-0628-1)
Supplement: Supplementary file 22 — (DOCX 21.7 kb) [file 13311_2018_628_MOESM19_ESM.docx]

**Supplementary Table 1.** **Clinical characteristics in 21 AD patients and 268 normal controls (youth group, middle-aged group and aged group).**

|  | | Youth | Middle-aged | Aged | AD | *p* value  (AD *vs* Aged) |
| --- | --- | --- | --- | --- | --- | --- |
| n | | 121 | 95 | 55 | 31 | NA |
| Age | | 25-45 | 46-60 | 60-90 | 50-91 | NA |
| Average age | | 31.84±0.46 | 51.02±0.42 | 73.00±1.22 | 69.65±1.65 | 0.11 |
| Gender | Male | 73 | 51 | 29 | 11 | NA |
|  | Female | 48 | 44 | 26 | 20 | NA |
| GLU(mmol/L) | | 5.17±0.06 | 5.21±0.04 | 5.17±0.07 | 6.31±0.31 | <0.01 |
| TC(mmol/L) | | 4.96 ±0.09 | 5.14±0.12 | 4.80±0.22 | 4.68±0.16 | 0.64 |
| LDL(mmol/L) | | 2.54±0.34 | 2.91±0.36 | 2.43±0.19 | 2.43±0.16 | 0.96 |
| HDL(mmol/L) | | 1.50±0.21 | 1.34±0.08 | 1.30±0.08 | 1.36±0.10 | 0.60 |
| TG(mmol/L) | | 1.17±0.06 | 1.37 ±0.09 | 1.30 ±0.11 | 1.35±0.10 | 0.76 |
