# Supplementary material for: Pigment Epithelium-Derived Factor Plays a Role in Alzheimer’s Disease by Negatively Regulating Aβ42
Source: Neurotherapeutics. 2018 May 7;15(3):728–41. doi: 10.1007/s13311-018-0628-1 (PMC6095778; doi:10.1007/s13311-018-0628-1)
Supplement: Supplementary file 23 — (DOCX 17.9 kb) [file 13311_2018_628_MOESM20_ESM.docx]

**Supplementary Table 2. Protective effects of PEDF on spatial memory of SAMP8 model mice.**

| Group N | | Latency at different time(s) | | | | |
| --- | --- | --- | --- | --- | --- | --- |
|  |  | Day1 | Day2 | Day3 | Day4 | Day5 |
| SAMR1+PBS | 12 | 50.25±4.55 | 45.14±6.03 | 48.09±9.00 | 36.99±5.35 | 40.90±7.47 |
| SAMP8+PBS | 12 | 64.85±6.31^*^ | 70.26±6.28^***^ | 64.81±7.72 | 67.62±5.04^***^ | 67.48±6.98^*^ |
| SAMP8+PEDF | 11 | 59.96±6.11 | 52.24±6.91 | 55.25±8.46 | 42.64±7.43^###^ | 43.09±6.94^#^ |

^*^*p*<0.05, ^***^*p*<0.001，vs SAMR1 normal control group at the same time;^#^*p*<0.05, ^###^ *p*<0.001, vs SAMP8 untreated group at the same time.
