# Supplementary material for: Pigment Epithelium-Derived Factor Plays a Role in Alzheimer’s Disease by Negatively Regulating Aβ42
Source: Neurotherapeutics. 2018 May 7;15(3):728–41. doi: 10.1007/s13311-018-0628-1 (PMC6095778; doi:10.1007/s13311-018-0628-1)
Supplement: Supplementary file 24 — (DOCX 13.2 kb) [file 13311_2018_628_MOESM21_ESM.docx]

**Supplementary Table 3. Swimming speed in escape latency.**

| Group N | | Swimming speed(cm/s) | | | | |
| --- | --- | --- | --- | --- | --- | --- |
|  |  | Day1 | Day2 | Day3 | Day4 | Day5 |
| SAMR1+PBS | 12 | 10.61±2.31 | 9.41±2.04 | 10.50±2.05 | 10.22±1.71 | 9.58±2.05 |
| SAMP8+PBS | 12 | 10.42±2.10 | 9.13±1.92 | 10.25±1.89 | 9.85±1.85 | 9.31±1.63 |
| SAMP8+PEDF | 11 | 10.69±1.65 | 9.38±2.02 | 10.34±2.41 | 9.87±1.77 | 9.31±1.68 |
