# Supplementary material for: Pigment Epithelium-Derived Factor Plays a Role in Alzheimer’s Disease by Negatively Regulating Aβ42
Source: Neurotherapeutics. 2018 May 7;15(3):728–41. doi: 10.1007/s13311-018-0628-1 (PMC6095778; doi:10.1007/s13311-018-0628-1)
Supplement: Supplementary file 25 — (DOCX 12.5 kb) [file 13311_2018_628_MOESM22_ESM.docx]

**Supplementary Table 4**

| Gene Primer sequences (5’ to 3’) |
| --- |
| *Ps1* Forward, AAGCGTATACCTAATCTGGGAGC  Reverse, TGCCGTTCTCTATTGTCATTCT  *β-actin* Forward, GCACTCTTCCAGCCTTCCTT  Reverse, GTTGG CGTACAGGTCTTTGC |
